# Insights into my experience as the Editor-in-Chief: a recap to close an unforgettable chapter

**DOI:** 10.1186/s13244-024-01878-3

**Published:** 2024-12-30

**Authors:** Luis Martí-Bonmatí

**Affiliations:** Medical Imaging Department and Biomedical Imaging Research Hospital Universitario y Politécnico La Fe and Health Research Institute, Valencia, Spain

Back in January 2018, I started my tenure leading the journal *Insights into Imaging* after being appointed by the ESR Board of Directors. However, my first interactions with the journal began much further in the past; when I contributed as a reviewer, I could have never imagined that one day I would be in the driver’s seat.

In these years leading the journal, the amount of work, learning, and satisfaction has been immense and has positively exceeded all my expectations. Being the Editor-in-Chief has been one of the greatest achievements in my career, and I am surely going to miss it. However, before I pass on the baton, I would like to (briefly) look back on this adventure.

One of my first wishes was to surround myself with an experienced and engaged Editorial Team to create a sense of culture that would define *Insights into Imaging*. I needed a strong group of Deputy Editors and an Editorial Board that was able to comprehensively cover the fields of radiological knowledge. Today, I feel immensely proud for having succeeded in this task by engaging a marvelous group of luminaries. This sense of culture was not only applicable to the Editorial Team but also to the Editorial Office. Throughout the years, we have fostered a close relationship that operates smoothly and has been critical to the growth of *Insights into Imaging*. I am deeply grateful for the years of professional and friendly collaboration.

It was important for me early on to place the journal’s focus on critical reviews, up-to-date evaluations, guidelines, evidence-based topics targeting the highest levels of evidence, insights into new developments (such as radiomics and artificial intelligence), professional issues, and management. To achieve this, I tried to adapt the editorial strategy of the journal and steer *Insights into Imaging* into becoming a journal for critical thinking with added clinical value. Only papers solving a gap in knowledge, improving a diagnostic pathway, or better explaining the criteria used to detect, characterize, or allocate treatments could pass the first Editor filter. Delivering patient-centric information requires training that is long, broad, and deep, and I have followed this strategy and these principles at all times to try to differentiate *Insights into Imaging* from other journals within the field of radiology [1].

The concept of ‘Critical Thinking’, which has been so deeply present during my editorship, aims to improve science by recognizing the uncertainties behind standard-of-care definitions, by questioning our understanding of what is and is not meaningful, and by identifying which are the biases of an established problem or pathway [2]. Specifically, in medical imaging, ‘Critical thinking’ deals with the process of evaluating efficacy from image acquisition to data processing and from biological correlation to therapeutic guidance. Through it, authors provide an insight into how radiology is currently practiced, searching for flaws through skeptical reasoning, while analyzing whether it needs to be re-shaped and, if so, how it must be done. We therefore aimed to receive submissions where previous knowledge, either eminence- or evidence-based, is critically reevaluated with a focus that would ultimately benefit patients [3]. In line with this, it is important to remember that skepticism is the first step toward truth; *Insights into Imaging* looks for manuscripts where different levels of evidence of existing knowledge are analyzed and questioned. In this scenario, ‘Evidence’ refers to the data, observations, or information that support a hypothesis or practical claim. ‘Evidence’ is continuously changing and evolving, following the common trends of new discoveries, advances in technology, replication and verification failures, and paradigm shifts [4]. Reflecting this in our published content was something we have continuously strived to achieve.

My attitude toward the editorial process was to always see it as an interactive discussion between authors, reviewers, and editors. I approached the authors’ work with the utmost respect, giving them detailed explanations for all the decisions I made and avoiding nonconstructive comments [1]. For the benefit of the authors, I used the fast desk rejections before peer review only when the topic or the study design was not within the scope of the journal or did not meet its requirements. This helped us avoid, as much as possible, delays in the first decision response but also saved valuable reviewer resources when it came to manuscripts that had a low probability of being accepted. Our reviewer pool was also one of the most important aspects of my time as Editor-in-Chief. Reviewer performance was carefully assessed and analyzed to help identify the top performers, who were later invited to join the Scientific Editorial Board or asked to contribute to the journal in other ways.

In order to increase our available pool of reviewers but also wishing to give back to the community, I was very proud of rolling out our ESR Journals Reviewer Workshop. After carefully evaluating the possibility of offering such a workshop, together with the European School of Radiology (ESOR), we held the very first edition of the workshop in Valencia in September 2024. The main goal of this workshop was to share knowledge and information between editorial members and reviewers, to create a common basis and understanding that will ultimately benefit the whole scientific community. I look forward to seeing it prosper in the future as a part of my legacy to *Insights into Imaging* and the whole ESR Journal Family.

It goes without saying that Artificial intelligence (AI) has been another main character during my time leading the journal. AI is the most impactful development in today’s radiology, medicine, science, and overall everyday life. To secure a reproducible result, faster translations into clinical practice, and efficient use of AI tools using radiomics, we have always encouraged authors to collect a sufficient sample size for the discovery, the internal test, and the independent external performance validation cohort to properly guarantee the reproducibility of the model and, therefore, to provide a clear clinical impact and a diagnostic gain over conventional approaches. We asked authors to describe how data was recruited, variables defined, and biases controlled.

Another milestone in my Editorship was the allocation of the journal’s first Impact Factor (IF). It was one of the highlights I experienced as it represents such an important metric. At the conclusion of my term of office, I am proud to say that we have achieved an IF that sets *Insights into Imaging* into the first quartile (Q1) of the journals’ category. This is a great success that underlines the relevance of all our Editorial policies and efforts. The positive IF has also affected the number of submissions to the journal (from 166 in 2018 to a projected 1756 in 2024) and, consequently, decreased the acceptance rate (from 61% in 2018 to a projected 18% in 2024).

Certainly, I cannot conclude this Editorial without declaring some of the ‘limitations’, tasks in the life of an Editor that were often more difficult and demanding than others: checking to detect plagiarism, reminding reviewers to submit their reviews, dealing with reviewers’ recommendations without specifying their reasons in the comments to the editor or even to the authors, carefully revising the reviewers’ comments on acceptance-rejection decisions in the reviews, and understanding reviewers’ recommendations when they were discordant with the comments in the review, just to name a few. Nevertheless, I am convinced that it was also those tasks that helped to shape the job that I have done with an incredible passion. All of them have enriched my understanding of scientific methodology, best imaging practices, and discovering professional pathways like no other task before.

It has been my absolute privilege to be a part of this world, which also allowed me to take part in the Editors Forum, an annual meeting together with the editors of several radiological journals. The team-building activities and the transparent discussions between peers have been a pivotal experience in understanding the role of the editors in keeping scientific journals as the doors of truth, innovation, and responsibility for authors and readers alike.

I am more than confident that Dr. Paola Clauser will continue the success story of *Insights into Imaging*, further improving the editorial process, increasing the quality of accepted articles, and advancing the international standing of the journal during her years as Editor-in-Chief. She has the full support of an excellent and solid team and my deepest admiration for undertaking this remarkable task.

Lastly, I want to bid farewell by saying that being the Editor-in-Chief of *Insights into Imaging* has been a continuous source of inspiration and powerful motivation for the huge responsibility that this role comes with. It has marked a truly unforgettable time in my career that I will forever cherish.
